# Boosted protease inhibitor monotherapy in HIV-infected adults: outputs from a pan-European expert panel meeting

**DOI:** 10.1186/1742-6405-10-3

**Published:** 2013-01-24

**Authors:** José R Arribas, Manuela Doroana, Dan Turner, Linos Vandekerckhove, Adrian Streinu-Cercel

**Affiliations:** 1 Consulta Medicina Interna 2, Hospital La Paz, IdiPAZ, Paseo de la Castellana 261, Madrid, 28046, Spain; 2 Serviço de Doenças Infecciosas, Hospital de Santa Maria, Av Prof Egas Moniz, Lisbon, 1649-035, Portugal; 3 Infectious Diseases Unit, Tel-Aviv Sourasky Medical Center, 6 Weizmann Street, Tel-Aviv, 64239, Israel; 4 AIDS Reference Centre, Ghent University Hospital, De Pintelaan 185, Ghent, 9000, Belgium; 5 HIV/AIDS Academy, Str Dr Calistrat Grozovici, Nr 1, Bucharest, 021105, Romania

## Abstract

While the introduction of combination highly active antiretroviral therapy (HAART) regimens represents an important advance in the management of human immunodeficiency virus (HIV)-infected patients, tolerability can be an issue and the use of several different agents may produce problems. The switch of combination HAART to ritonavir-boosted protease inhibitor (PI) monotherapy may offer the opportunity to maintain antiviral efficacy while reducing treatment complexity and the risks of toxicity. Current European AIDS Clinical Society (EACS) guidelines recognise ritonavir-boosted PI monotherapy with twice-daily lopinavir/ritonavir or once-daily darunavir/ritonavir as a possible option in patients who have intolerance to nucleoside reverse transcriptase inhibitors, or for treatment simplification. Clinical trials data for PI boosted monotherapy are encouraging, showing substantial efficacy in the majority of patients; however, further data are required before this approach can be recommended as a routine treatment. Available data indicate that the most suitable candidates for the use of boosted PI monotherapy are long-term virologically suppressed patients who have demonstrated good adherence to antiretroviral therapy, who do not have chronic hepatitis B, have no history of treatment failure on PIs and are able to tolerate low-dose ritonavir.

## Introduction

**Figure 1:**
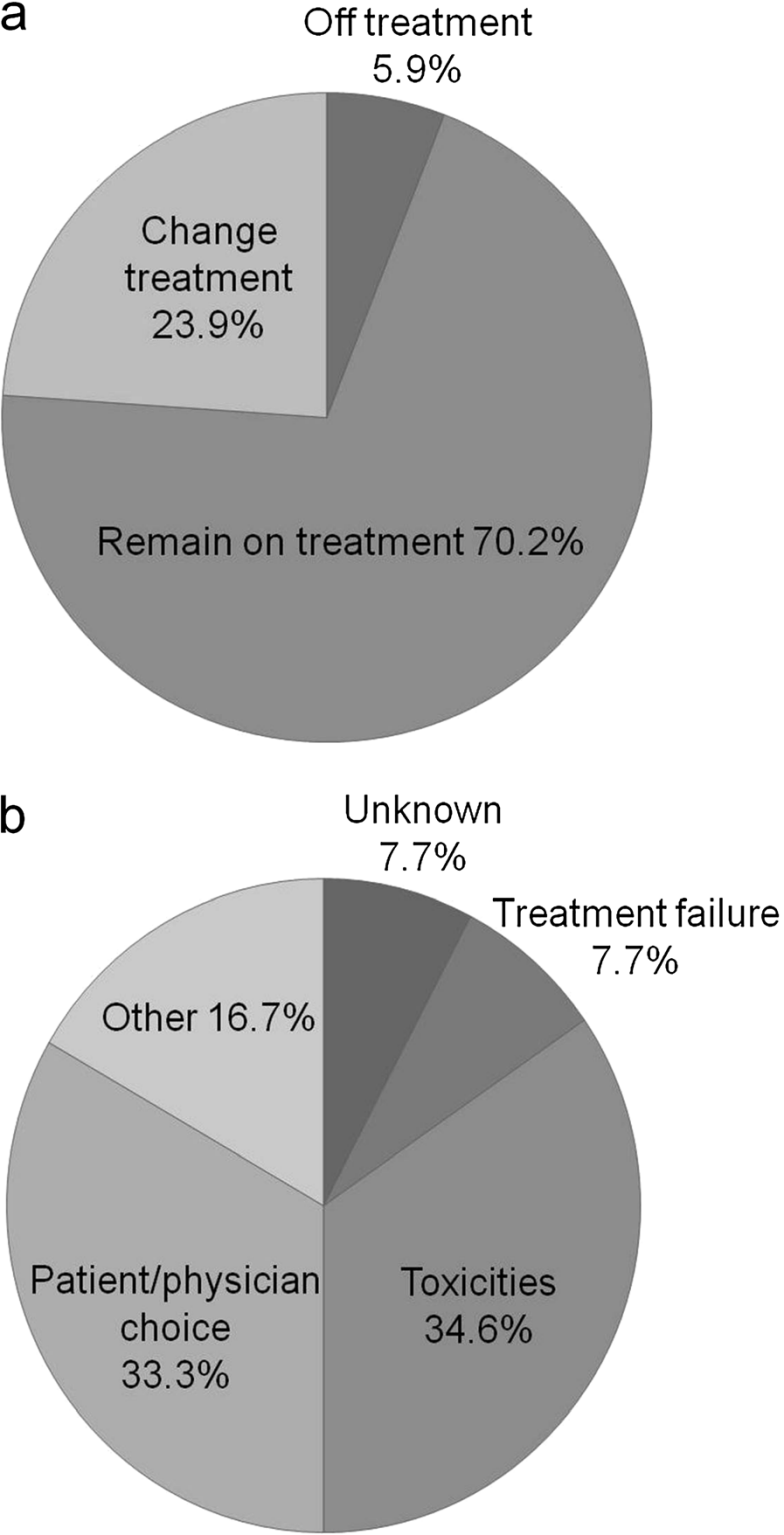
**a) Proportion of patients remaining on original treatment regimen 1 year after initiating HAART; b) Reasons for discontinuing HAART between 2002–2004 **[1]**.**

While the introduction of highly active antiretroviral therapy (HAART), typically involving three drugs in combination, has been an important advance in the treatment of people infected with human immunodeficiency type-1 virus (HIV-1), many patients do not remain on their original treatment regimen one year after starting HAART (EuroSIDA study; Figure 1) [1]. Common reasons for discontinuing HAART in chronically treated patients include patient/physician choice, treatment failure and tolerability issues such as renal and cardiovascular toxicity with nucleoside reverse transcriptase inhibitors (NRTIs), and, with the non-NRTIs (NNRTIs), dyslipidaemia with efavirenz and rash with nevirapine. Important developments for the future of HAART are to improve its potency and activity against multi-drug resistance viruses, to improve dosing schedules and their convenience, and to improve the tolerability of treatment.

It is relatively common for treatment regimens to be switched in fully suppressed HIV-1-infected patients. These regimen switches can be either proactive (to avoid potential problems) or reactive (in response to poor tolerability or other issues). For example, in pregnant women the regimen may be switched to avoid risk of teratogenicity and to optimise pharmacokinetics, while in other patients regimens may be switched to improve adherence or convenience, to preserve future treatment options, to avoid drug interactions or to reduce costs. Maintenance of virological control is paramount when switching therapies, and treatment switches in virologically suppressed patients was considered by the panel members to be one of the most common therapeutic interventions in HIV therapy in late 2011.

While intermittent therapy, sequential or prolonged treatment interruptions are no longer recommended as switch strategies by HIV treatment guidelines, [2] there remains a variety of switch strategies for use in suppressed patients to deal with toxicity, to simplify treatment regimens and to try to meet patients’ wishes for their treatment. These include reducing pill burden, ritonavir-sparing and so-called ‘nuc’ (NRTI)-sparing regimens. Among the various switching options available, the strategy of switching from a triple-drug regimen to ritonavir-boosted protease inhibitor (PI/r) monotherapy is currently a topic of some interest as it offers a number of potential benefits, including a reduction of NRTI-related toxicity (lipoatrophy, renal disease, bone mineral density [BMD] loss) and reduced cost versus dual or triple therapy while retaining other classes as future treatment options. This review provides an overview of the use of boosted PI monotherapy in Europe and the potential benefits and drawbacks of such therapy.

## EACS 2011 guidelines on treatment switch strategies

The current European AIDS Clinical Society (EACS) guidelines for the management of HIV-infected patients list three principal indications for the use of switch strategies in virologically suppressed patients (plasma viral load < 50 HIV-1 RNA copies/mL) [2]:

•switch for toxicity – including documented toxicity, the management of potential drug interactions, side effects, and planned pregnancy

•switch for the prevention of long-term toxicity – including pre-emptive switching; ageing and/or co-morbidity with a possible negative impact of drug(s) in the current regimen (e.g. on cardiovascular risk and metabolic parameters)

•switch for simplification – including the wish to simplify the regimen or because the treatment regimen is no longer recommended.

The EACS 2011 guidelines state that PI/r monotherapy with lopinavir twice daily or darunavir once daily might represent an option in those patients intolerant to NRTIs, or for treatment simplification [2]. The guidelines note that this strategy only applies to patients without a history of failure on prior PI-based therapy and who have had viral load < 50 HIV-1 RNA copies/mL in at least the past 6 months. In considering a switch to boosted PI monotherapy, attention must also be given to the duration of virological suppression prior to the considered switch, to any pharmacokinetic/dynamic considerations, to the expected level of adherence and to the individual patient’s needs. Boosted PI monotherapy should not be prescribed to patients co-infected with chronic hepatitis B.

In the USA, PI/r monotherapy is not recommended by the Department of Health and Human Services (DHHS) 2011 guidelines outside of the clinical trial setting, [3] and PI/r monotherapy is only recommended by the International AIDS Society-USA (IAS-USA) 2010 guidelines in exceptional circumstances when other drugs cannot be considered for reasons of toxicity/tolerability. Other treatment guidelines offer differing recommendations with respect to boosted PI monotherapy. At present only the EACS, [2] Italian [4] and Spanish Gesida guidelines [5] recognise PI/r monotherapy as a switch option in the everyday clinical setting. There is currently a lack of consensus in the treatment guidelines with respect to monotherapy. However, this situation may change as the results of ongoing clinical studies of PI/r monotherapy become available.

## Current use of boosted PI monotherapy in Europe

At present, there is some variation in clinical practice with regard to the use of boosted PI monotherapy between European countries and regions (e.g. Italy where monotherapy tends to be only used at the larger treatment centres). It is thought there are several reasons for this variation. Some clinicians consider the number of patients included in monotherapy studies to be too few for drawing valid conclusions. It has been suggested that some clinicians could be detracted from initiating boosted PI monotherapy by potential legal implications. Finally, geographic variation may also arise from physicians being influenced by US guidelines and the presence of pre-existing PI resistance.

## Reasons for variability in the use of boosted PI monotherapy in Europe

Switching from triple-drug HAART regimens to boosted PI monotherapy offers benefits in terms of reduced complexity, the potential for a reduction in some toxicities (though longer-term data are required to more fully assess the tolerability profile of PI/r monotherapy versus HAART), and in terms of cost savings. However, as with any new treatment approach, there are a number of potential issues and concerns about boosted PI monotherapy and such concerns may at least in part account for the observed differences in the adoption of this approach across different European countries. There is evidence that boosted PI monotherapy for maintenance of viral suppression is less effective than triple-drug HAART [6]. However, the observed difference in efficacy between HAART and PI/r monotherapy is small and differences may be PI dependent. Furthermore, although there is an increased risk of low-level viraemia with monotherapy in a subgroup of patients, this has been shown to be reversible after NRTI reintroduction. Clinical trials with darunavir/r [7-9] and lopinavir/r [10] monotherapy have not shown an increased risk of resistance development.

Another potential drawback of PI/r monotherapy is that such regimens are ‘less forgiving’ of poor adherence than triple-drug regimens, so that a high level of adherence is required for patients switching to PI/r monotherapy. In this regard, clinical trials have shown an association between suboptimal adherence and virological failure in patients treated with lopinavir/r [11,12] or darunavir/r monotherapy [13]. It has been hypothesised that suboptimal adherence may have a greater effect on virological suppression in patients receiving boosted PI monotherapy than it does in those on combination therapy including nucleosides due to the short terminal half-lives of lopinavir and darunavir compared with the long intracellular half-lives of nucleosides [10,14]. It is in any case clear that, in addition to their having had a sufficient period of virological suppression on HAART (at least 6 months), candidates for PI/r monotherapy should demonstrate a history of good adherence to prescribed treatments. In acknowledging the importance of good treatment adherence for the success of boosted PI monotherapy it should be remembered that, as shown in clinical trials, patients failing PI/r monotherapy can be successfully treated with the re-introduction of nucleosides. In this regard, it is important that patients receiving PI/r monotherapy are regularly monitored to allow early detection of ‘reversible failures’.

### Selection of patients for boosted PI monotherapy

Perhaps the most pertinent question in considering the initiation of PI/r monotherapy is: who are the best candidates and how can we best identify them? The selection of patients and the subsequent demonstration of successful long-term PI/r monotherapy is likely to be critical in the consolidation of monotherapy as an accepted treatment approach. The authors of the MONOI study concluded that good candidates for PI/r monotherapy should have an undetectable viral load (HIV-1 RNA at least < 50 copies/mL) at the time of commencing monotherapy, should be more adherent than for triple therapy and should have been treated with antiretroviral therapy (ART) for a sufficient amount of time before switching to monotherapy [15].

While it is clear that it is important for PI/r monotherapy candidates to have a substantial period with an undetectable viral load, opinions differ with regard to the minimum duration of viral suppression. Most switching studies have used a 6-month period with undetectable viral load, primarily in order to demonstrate that existing therapy was effective prior to the switch and that the patient was adherent. Adherence to treatment is important, and patients who have been stable on HAART for some years and who have shown no signs of poor compliance would appear to be suitable, provided that there are no other factors that would preclude PI/r monotherapy, such as a history of PI failure. With regard to adherence, in MONOI, 48.5% of rebounding PI/r monotherapy patients admitted to having missed at least one treatment dose during follow-up versus 31.6% of other patients [15]. The median duration of prior ART treatment in rebounding PI/r monotherapy patients was 5.1 years versus 8.9 years in other patients.

With regards to pre-HAART HIV DNA, in MONOI the median HIV DNA was 4.2 log_10_ copies/10^6^ cells in patients with virological rebound during 96 weeks of darunavir/r monotherapy (24/112 patients, 21.4%) versus 3.9 log_10_ copies/10^6^ cells in other patients [15]. Furthermore, it was demonstrated (using ultrasensitive plasma RNA assay and quantification of total HIV DNA viraemia) that patients in the MONOI study with the lowest plasma RNA and total HIV DNA viraemia were the most suitable candidates for PI/r monotherapy [13,15].

CD4 cell count status is also an important factor when considering PI/r monotherapy initiation. Analyses to predict PI/r monotherapy failure have shown a significant association between a low baseline CD4 cell count nadir (< 100–200 cells/μL) and subsequent treatment failure [11,16]. Furthermore, in patients co-infected with hepatitis C virus, significantly lower virological response was observed versus non-co-infected patients [17], suggesting that hepatitis C co-infection, likely associated with intravenous drug use, might possibly be a marker of poor adherence**.**

### Maintaining virological efficacy with boosted PI monotherapy

A key consideration for the adoption of boosted PI monotherapy is to achieve a consensus on what constitutes treatment success. It is also important to demonstrate that effective viral suppression can be maintained over the long term. It is worth noting that even with combination therapy, the current ‘gold standard’ for achieving durable viral suppression, isolated ‘blips’ (i.e. viral loads that are transiently detectable, but usually no more than 400 HIV-1 RNA copies/mL), are not uncommon in successfully treated patients, so some degree of fluctuation in viral load might also be expected with PI/r monotherapy. In addition to effective viral suppression, the success of treatment must also be defined in terms of its tolerability for the patient and the balance of efficacy/tolerability achieved in relation to other possible treatment approaches. While the success of triple-drug HAART in achieving viral suppression is clear, it is also clear that the long-term success of HAART has, in part, been limited by drug toxicities and drug interactions, as patients are exposed to multiple drugs for prolonged periods of time [18,19].

Although plasma HIV RNA < 50 copies/mL is today consistently considered as undetectable, treatment success has been defined differently across clinical trials e.g. different primary and secondary endpoints providing different power calculations and levels of confidence, as well as different analysis definitions (intent-to-treat [ITT]-time-to-loss of virological response [TLOVR] and TLOVR non-virological failure [VF] censored etc.). It is to be hoped that future studies will adopt similar criteria to assess efficacy and safety parameters for treatments to make it easier to compare their findings. Ideally, treatment decisions would be informed by access to long-term data from controlled clinical trials and cohort studies, but such data are unavailable for most individual treatments and regimens.

Defining treatment failure also remains challenging, with different guidelines offering different perspectives based on different evidence. The EACS guidelines define VF as a confirmed plasma HIV-1 RNA > 50 copies/mL six months after starting therapy (initiation or modification) in patients remaining on ART [2]. In contrast, the US DHHS guidelines define VF as a confirmed viral load > 200 HIV-1 RNA copies/mL, on the basis that it eliminates most cases of apparent viraemia caused by blips or assay variability [3]. However, given the range of viral load assays available, with different sensitivities, it remains possible that different results may be obtained with different assays, such that a patient might be judged to be ‘borderline’ but still successfully treated using one assay, but a ‘failure’ requiring consideration of a change in treatment using another assay.

**Figure 2:**
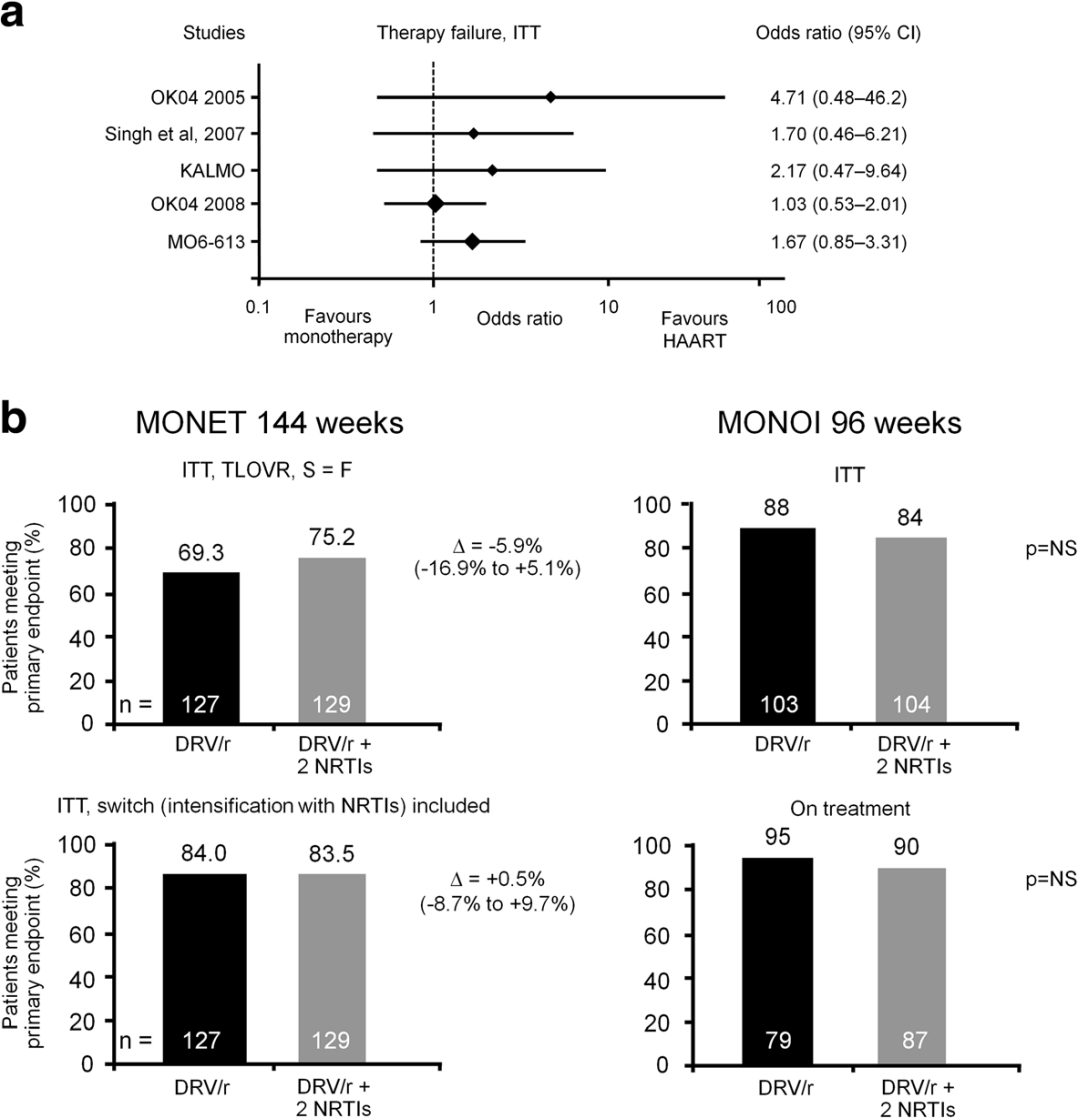
**Efficacy of a) Lopinavir/r **[21]**and b) Darunavir/r monotherapy vs comparator arms **[7,20]**.** Efficacy was assessed in the ITT population and modification of treatment was considered failure in the MONET and MONOI darunavir/r monotherapy trials [7,20]. In the lopinavir/r monotherapy trials [21], there was some variance in what was considered failure. In OK04 2005, treatment modification = failure. In OK04 2008, treatment modification ≠ failure. In KALMO failure was defined as confirmed HIV-1 RNA > 1,000 copies/mL. In MO6-613 non-responders were defined as those who either did not complete 96 weeks of treatment, had HIV-1 RNA at > 50 copies/mL at week 96, or experienced confirmed virological rebound before week 96.

While boosted PI monotherapy offers the potential for reduced toxicity, its adoption depends upon the demonstration of robust efficacy data. Available data show only a small difference in efficacy between triple-drug HAART and boosted PI monotherapy following treatment switch (Figure 2a [lopinavir/r] and 2b [darunavir/r]) and in the MONET study with darunavir/r monotherapy, effective viral suppression was maintained up to three years of treatment [20]. Of note, in the five patients with VF on darunavir/r monotherapy in the MONOI study, re-suppression of viral load (< 50 HIV-1 RNA copies/mL) was successfully achieved with the addition of two NRTIs to the treatment regimen. The emergence of PI resistance on PI/r monotherapy has been rare and re-introduction of HAART remains possible in such cases. It is also important to note that in the PI monotherapy trails reviewed, changes in CD4 cell counts between treatment arms were not considered significant [7,20,21]. In their systematic review of boosted PI monotherapy, Bierman *et al*. reported that mean CD4 cell counts ranged from −40 to +289 cells/μl in patients on monotherapy against +8 to +240 cells/μl in patients on HAART [21]. In summary, the available evidence indicates that, in a large proportion of patients, effective long-term virological control is possible with boosted PI monotherapy and if re-suppression is needed this can be achieved with the re-introduction of NRTIs.

### The significance of detectable virus in sanctuary sites

While modern ART can provide effective plasma HIV-1 virus suppression, the importance of viral reservoirs in the genitals and central nervous system (CNS) remains the subject of debate. The principal issues surrounding these reservoirs are whether or not three drugs are still required to control HIV-1 replication in sanctuary sites when plasma HIV-1 RNA is fully suppressed, whether all boosted PIs sufficiently penetrate into the CNS to achieve effective long-term control, and whether boosted PI monotherapy controls HIV-1 RNA in the genital tract. Antiretroviral drug penetration may be important for preventing HIV-1 replication in the CNS, and observational studies have demonstrated a correlation between CNS penetration effectiveness rank (CPE) and HIV-1 levels in the cerebrospinal fluid (CSF) [22]. Further, darunavir/r and lopinavir/r monotherapy have lower CPE scores compared with triple therapy regimens [23]; although it should be noted that CPE score has yet not been validated.

It is thought that the presence of HIV-1 in the CNS results in neurotoxicity [23], with a number of clinical factors associated with its development, including depression, female gender, low CD4 count, advancing age and drug abuse [24,25]. Consequently, an important question is to what degree plasma viral suppression to < 50 HIV-1 RNA copies/mL translates into effective viral suppression in the CNS. It has been suggested that CSF viral load data could be obtained as part of the decision to initiate therapy. In this regard, in the CHARTER study, 96% of patients with undetectable plasma HIV-1 RNA on a triple regimen also had a suppressed CNS viral load [23], indicating that most patients on HAART have effective suppression of HIV-1 in the CNS.

Conversely, since NRTIs potentially have adverse effects on the CNS, monotherapy with boosted PIs have the possible advantage to the CNS of removal of NRTI toxicity. The NRTIs are known to penetrate the CNS and have been associated with improvements in cerebral function. However, administration of the NRTIs can also result in mitochondrial toxicities through CNS penetration. Indeed, several NRTIs have been shown to possess the potential to damage brain tissue and neurones [26,27]. Hence, the use of PI/r monotherapy could decrease some of the CNS toxicities associated with combination therapy.

The current EACS guidelines define potentially CNS-active drugs as antiretrovirals with either demonstrated clear CSF penetration when studied in HIV-infected populations (CSF concentration above the 90% inhibitory concentration [IC_90_] in > 90% of examined patients), or with proven short-term (3–6 months) efficacy with respect to cognitive function or CSF viral load decay when evaluated as single agents or in controlled studies in peer reviewed papers. According to this definition, agents with demonstrated clear CSF penetration are the NRTIs zidovudine and abacavir, the NNRTIs efavirenz and nevirapine, the boosted PIs darunavir/r, lopinavir/r and indinavir/r, and the entry inhibitor maraviroc, while agents with ‘proven efficacy’ are the NRTIs zidovudine, stavudine and abacavir, and the boosted PI lopinavir/r [2].

With regard to the genital reservoir for HIV, in a MONOI sub-study investigating the penetration of darunavir into seminal fluid, darunavir seminal plasma concentration was 6-fold higher than the darunavir 50% effective concentration (EC_50_) against wild-type HIV-1, with the median concentration close to the blood plasma free fraction [28]. In females, in the IMANI-2 study of lopinavir/r first-line monotherapy, lopinavir/r penetration into the cervicovaginal fluid exceeded the reference population median IC_50_ value in all but one sample tested [29].

The available data indicate that both darunavir and lopinavir show moderately good CNS penetration, with both showing high penetration into the genital tract. However, further studies are needed to confirm these findings.

### Safety and tolerability benefits with boosted PI monotherapy

**Figure 3:**
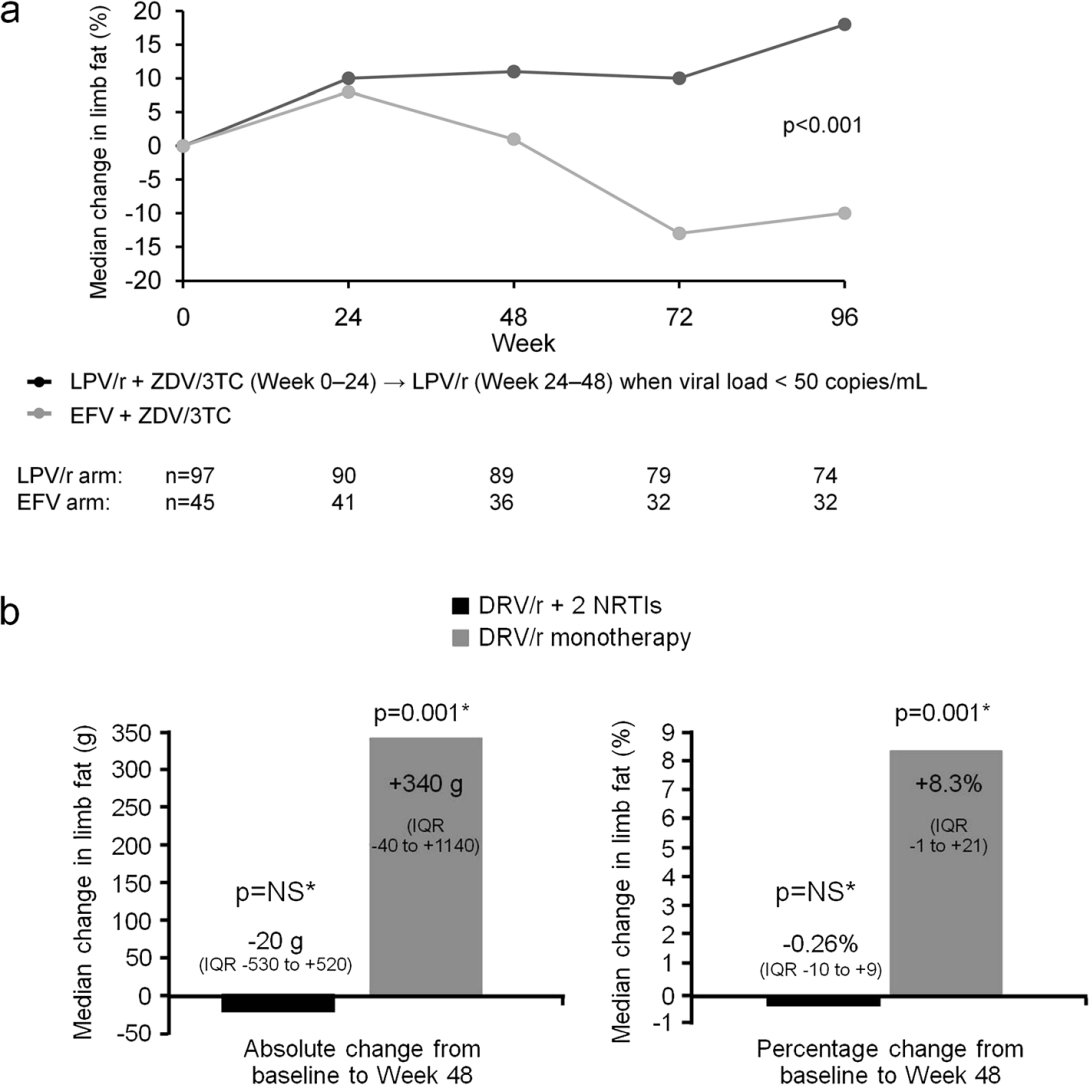
**a) Abbott 613 trial:** Changes in limb fat over time [30]**b)** MONOI: Median change in limb fat from baseline to week 48 [31]**.**

One of the main attractions of a switch from multi-drug HAART to boosted PI monotherapy is the potential safety benefit that may result from its use. The tolerability issues of NRTIs in particular are well known, these agents being associated with increased risk of insulin resistance, secondary dyslipidaemia and lipodystrophy/atrophy. Reduced BMD and renal disease may also result with some HAART regimens. Lipodystrophy seems to be associated with particular NRTIs, with regimens containing stavudine or zidovudine appearing to confer highest risk [30]. Data from the M03-613 study in treatment-naïve patients taking lopinavir/r (Figure 3a) and the MONOI trial with darunavir/r (Figure 3b) indicate that boosted PI monotherapy is not associated with the limb fat loss that is observed with some NRTIs [31,32], although longer-term tolerability data are required to confirm the findings.

When considering BMD, while there was a slightly greater decrease in BMD in lopinavir/r monotherapy-treated patients versus triple-therapy patients over 96 weeks in the M03-613 study, in the 48-week MONARCH study in 30 virologically suppressed patients, the switch to darunavir/r monotherapy was associated with a trend for improvement in BMD (and in body fat distribution with stable limb fat and a decrease in visceral adipose tissue) [33]. While long-term data are required, available data indicate that switching to boosted PI monotherapy does not adversely affect BMD. It should be noted, however, that there were greater increases in total and low density lipoprotein cholesterol with PI/r monotherapy versus triple therapy in MONARCH, though this may reflect the fact that some patients in the triple therapy arm were taking tenofovir, which has a direct lipid-lowering effect [34].

Renal toxicity is a well-recognised risk with tenofovir disoproxil fumarate therapy [35]. From this perspective, boosted PI monotherapy would appear to be a useful treatment option for patients with tenofovir-related renal disease. Finally, while concerns have been expressed about potential CSF viral escape with PI/r monotherapy, after 144 weeks of darunavir/r monotherapy in the MONET study there was no significant difference in the observed change in cognitive functioning between the mono- and triple therapy treatment groups (although the study was not powered to detect differences in neurologic impairment) [36].

## Future development of boosted PI monotherapy: ongoing and planned studies

While available data indicate that the switch from multi-drug HAART to boosted PI monotherapy in suitable patients represents an attractive treatment option, there remain some questions to be answered, in particular relating to long-term efficacy and tolerability, and efficacy in CNS and genital ‘reservoirs’ (although in the latter case this is also true for triple therapy).

Two ongoing studies of boosted PI monotherapy are expected to provide a wealth of new efficacy and tolerability data: PIVOT (PI monotherapy Vs Ongoing Triple-therapy in the long-term management of HIV infection) and PROTEA, (PROTEAse inhibitor [darunavir/r] in mono- or triple therapy in suppressed HIV-1-infected patients). The PIVOT trial planned to include 560 patients in the UK and be of 5 years’ duration. Patients with a plasma viral load < 50 HIV-1 RNA copies/mL at screening (and for at least 24 weeks prior to screening) and a CD4 cell count > 100 cells/mm^3^ are included in the study, which compares boosted PI monotherapy to combination HAART with two NRTIs and either an NNRTI or a PI (boosted or un-boosted). This study, which includes genital secretions and CNS sub-studies, will also measure effects on quality of life, neurocognitive function, cardiovascular risk and costs.

The phase IIIb PROTEA study is investigating the efficacy and tolerability of darunavir/r monotherapy versus darunavir/r-containing triple therapy in approximately 260 virologically suppressed (for at least 48 weeks) HIV-1-infected patients. The primary objective is to demonstrate the non-inferior efficacy of darunavir/r monotherapy (with respect to the percentage of patients with viral load < 50 HIV-1 RNA copies/mL at 48 weeks post-switch to monotherapy) versus triple therapy containing darunavir/r. This study will also assess changes in neurocognitive function over 48 and 96 weeks of treatment and whether there is any correlation between plasma and CSF viral load and neurocognitive function. In addition, PROTEA will study the evolution of the viral genotype and determine whether there is any loss of treatment options at weeks 48 and 96, as defined by the emergence of phenotypic drug resistance.

## Summary and conclusions

The switch from triple-drug combination therapy to boosted PI monotherapy appears to be a promising strategy to maintain antiviral efficacy. Available data from PI/r monotherapy trials are encouraging, showing slightly lower efficacy than triple therapy regimens. However, further data – particularly with regard to the long-term efficacy and tolerability of treatment – are required before this approach can be recommended as a routine treatment option. In this regard, further studies are ongoing, which will further elucidate the long-term utility of boosted PI monotherapy. In the meantime, the available data indicate that the most suitable candidates for the use of boosted PI monotherapy are long-term virologically suppressed patients who have demonstrated good adherence to ART, have no history of PI failure, do not have chronic hepatitis B, do not have HIV-associated neurocognitive disorders and who are able to tolerate low dose ritonavir.

## Abbreviations

ART: antiretroviral therapy; BMD: bone mineral density; CNS: central nervous system; CPE: CNS penetration effectiveness rank; CSF: cerebrospinal fluid; DHHS: Department of Health and Human Services; EACS: European AIDS Clinical Society; EC: effective concentration; HAART: highly active ART; HIV: human immunodeficiency virus; IAS-USA: International AIDS Society-USA; IC: inhibitory concentration; ITT: intent-to-treat; NNRTI: non-nucleoside reverse transcriptase inhibitor; NRTI: nucleoside reverse transcriptase inhibitor; PI: protease inhibitor; TLOVR: time to loss of virological response; VF: virological failure.

## Competing interests

Professor JR Arribas has received advisory fees, speaker fees and grant support from the following sources: Abbott, Bristol-Myers Squibb, Gilead, Janssen, MSD, Tibotec and ViiV Healthcare. Dr M Doroana has received speaker fees from different sources: Gilead, Roche, Janssen and Abbott. Dr D Turner has received honoraria from the following sources: Abbott, Biotis, GlaxoSmithKline, Janssen, MSD, Neopharm, Roche and Schering-Plough. Professor L Vandekerckhove has received advisory fees, speaker fees and grant support from the following sources: Gilead, Janssen, MSD, Bristol-Myers Squibb, Pfizer and ViiV Healthcare. Professor A Streinu Cercel has relationships with all major pharmaceutical companies but without any specific arrangements.

## Authors’ contributions

All authors substantially contributed to development of all drafts of the manuscript and have read and approved the final draft. The corresponding author had full access to the source literature and takes full responsibility for the content of the paper and for the decision to submit.

## Authors’ information

Panel members: Professor José Arribas (Spain); Professor Giovanni Di Perri (Italy); Professor Pierre-Marie Girard (France); Dr Alan Winston (UK); Professor Hans-Jürgen Stellbrink (Germany); Professor Adrian Streinu-Cercel (Romania); Professor Linos Vandekerckhove (Belgium); Professor Adriano Lazzarin (Italy); Dr Dan Turner (Israel); Dra Manuela Doroana (Portugal); Dr Esteban Martinez (Spain); Dr Markus Bickel (Germany); Dr Panagiotis Kollaras (Greece); Dr George Panos (Greece); Dr Giovanni Guaraldi (Italy); Professor Sorin Rugina (Romania); Professor Carmen Dorobat (Romania); Dr Dan Duiculescu (Romania).
